# Structural, Magnetic, and AC Measurements of Nanoferrites/Graphene Composites

**DOI:** 10.3390/nano12060931

**Published:** 2022-03-11

**Authors:** Shaimaa A. Habib, Samia A. Saafan, Talaat M. Meaz, Moustafa A. Darwish, Di Zhou, Mayeen U. Khandaker, Mohammad A. Islam, Hamidreza Mohafez, Alex V. Trukhanov, Sergei V. Trukhanov, Maha K. Omar

**Affiliations:** 1 Physics Department, Faculty of Science, Tanta University, Tanta 31527, Egypt; shaimaa.habib@sci.dmu.edu.eg (S.A.H.); samiasaafan@science.tanta.edu.eg (S.A.S.); tmeaz@science.tanta.edu.eg (T.M.M.); mostafa_ph@science.tanta.edu.eg (M.A.D.); maha.omr@science.tanta.edu.eg (M.K.O.); 2 Physics Department, Faculty of Science, Damnhour University, Damanhour 22516, Egypt; 3 Electronic Materials Research Laboratory, Key Laboratory of the Ministry of Education & International Center for Dielectric Research, School of Electronic Science and Engineering, Xi’an Jiaotong University, Xi’an 710049, China; zhoudi1220@gmail.com; 4 Centre for Applied Physics and Radiation Technologies, School of Engineering and Technology, Sunway University, Petaling Jaya 47500, Malaysia; mayeenk@sunway.edu.my; 5 Department of Electrical Engineering, Faculty of Engineering, Universiti Malaya, Kuala Lumpur 50603, Malaysia; aminul.islam@um.edu.my; 6 Department of Biomedical Engineering, Faculty of Engineering, Universiti Malaya, Kuala Lumpur 50603, Malaysia; 7 Department of Electronic Materials Technology, National University of Science and Technology MISiS, 119049 Moscow, Russia; truhanov86@mail.ru; 8 Laboratory of Magnetic Films Physics, SSPA “Scientific and Practical Materials Research Centre of NAS of Belarus”, 19, P. Brovki Str., 220072 Minsk, Belarus; 9 Laboratory of Single Crystal Growth, South Ural State University, 76, Lenin Av., 454080 Chelyabinsk, Russia

**Keywords:** spinel ferrites, graphene, composites, magnetic properties, AC conductivity

## Abstract

As a contribution to the graphene-based nanoferrite composites, this article is intended to present Mn, Co, and Co-Mn nanoferrites for the preparation and investigation of such samples. Nanoparticles of Co ferrite, Mn ferrite, and Co-Mn ferrite were chemically synthesized by the coprecipitation method. The composites of ferrite/graphene were made by incorporating weight ratios of 25% graphene to 75% ferrite. Various structural and characterizing investigations of ferrite samples and ferrite/graphene composites were performed, including XRD, EDX, SEM, VSM hysteresis loops, AC conductivity, and dielectric behavior. The investigations ensured the formation of the intended nanoferrite powders, each having a single-phase crystal structure with no undesired phases or elements. All samples exhibit a soft magnetic behavior. They show a semiconducting behavior of AC electrical conductivity as well. This was proved by the temperature dependence of the AC’s electrical conductivity. Whereas the dielectric function and loss tangent show an expected, well-explained behavior, the ferrite/graphene composite samples have lower saturation magnetization values, lower AC conductivity, and dielectric constant values than the pure ferrites but still have the same behavior trends as those of the pure ferrites. The values obtained may represent steps on developing new materials for expected applications, such as manufacturing supercapacitors and/or improved battery electrodes.

## 1. Introduction

Over the last few decades, the global need for energy has skyrocketed. Nonrenewable fossil fuels, whose reserves will deplete in the future, account for the majority of the increase in global energy use, not to mention that those sources are not environmentally friendly. As a result, new energy systems utilizing renewable energy sources are urgently required. For electric automobiles and other extensively utilized energy storage devices, there are many materials, so science researchers are working on new battery and supercapacitor technologies [1]. The electrode materials employed determine the capacitance and storage capacity of supercapacitors. Supercapacitors have a distinct role in current technology because they bridge the gap between batteries and ordinary capacitors by providing stored energy quickly and effectively [2]. While Li-ion batteries are already widely used in electric devices and cars, the need for new battery technologies beyond Li-ion batteries has drawn the scientific community’s attention.

Research into new materials and an in-depth understanding of the science behind these devices, such as redox processes, electrode−electrolyte interaction, and cell-component aging, are necessary to build high-performance battery systems. Electrode materials can be evaluated by identifying their chemical composition, crystal structure, and electrical configuration as well as monitoring their attributes during electrochemical cell operation [3]. Recently, graphene-based materials such as ferrite/graphene composites have emerged as new materials for many critical applications [4,5,6,7,8], especially for energy storage. These composites may provide promising candidates for both supercapacitors [9,10,11,12,13] and battery systems [14,15,16]. Also, using graphene, reduced graphene oxide, carbon fibers, multiwall carbon nanotubes, etc. for preparing composite materials are promising for electromagnetic shielding and wastewater treatment applications [17,18,19] and have many different potentials and environmentally friendly applications [20,21,22,23].

It is worth noting that ferrites—which are iron-containing complex oxides—are critical magnetic materials in business and engineering due to their widespread use in electronics and communication technology. Many of those applications have involved the usage of structured spinel ferrites with the general formula MeFe_2_O_4_ (where Me is a transition metal such as Ni, Cu, Mn, Fe, or Co). The characteristics of ferrite can be significantly altered by altering the divalent Me^2+^ cation and/or the technique of production [24,25].

Due to its unique features, as well as its practical applications, graphene and graphene-based materials have drawn the interest of researchers. Additionally, graphene may be used to form composites with a wide range of organic and inorganic functional components, making it a versatile material. The surface-to-volume ratio of a magnetic ferrite/graphene nanocomposite may be ideal for some key interactions and reactions. Additionally, the ferrites’ magnetic properties and the electrical conductivity of the graphene sheets in these heteroarchitectures show a unique electrochemical behavior [26].

It is worth noting that spinel ferrites have been suggested as an anode material for lithium-ion batteries (LIBs) to boost their energy density. Even yet, it has been discovered that in charge/discharge processes, pure CuFe_2_O_4_ electrodes have low intrinsic electrical conductivity and large volume changes; they also have a small capacity and poor cycling stability. These nanoparticles of transition metal oxides can be combined with carbon-based materials to improve ferrites’ electrochemical performance as anode materials [25].

Therefore, the present work aims to synthesize, characterize, and investigate the properties of nanoparticle ferrite/graphene composites, which are expected to be good candidates for the electronics industry and energy storage applications.

## 2. Materials and Methods

### 2.1. Ferrite Nanoparticles Synthesis

Three chemical compositions, CoFe_2_O_4_, MnFe_2_O_4_, and Co_0.5_Mn_0.5_Fe_2_O_4_, were chemically prepared by the coprecipitation method. The metal chlorides (CAS 1313-99-1, Luoyang Tongrun Nano Technology Co., Ltd., Luoyang, Henan, China) were used as the starting precursors along with NaOH (CAS 1313-99-1, Luoyang Tongrun Nano Technology Co., Ltd., Luoyang, Henan, China). The prepared technique was published before in detail [27]. Some amounts of the synthesized powders were used for X-ray diffraction (XRD) (Rigaku Europe SE, Neu-Isenburg, Germany), energy dispersive X-ray (EDX) (Oxford Instruments, Moscow, Russia), scanning electron microscopy (SEM) (Lyra3, Tescan, Brno, Czech Republic), and vibrating sample magnetometer (VSM) (Criogenic Ltd., London, United Kingdom) measurements, and some other amounts were pressed into small pellets for electrical measurements.

### 2.2. Structure Investigation

At first, the crystal formation and the size of crystallites of the pure ferrites were investigated by an XRD instrument. The samples were further investigated by an EDX and SEM.

### 2.3. Preparation of the Composites

The graphene was bought from a specialized chemical company (Nano Gate, Cairo, Egypt), where they use a well-known method of preparing graphene [28]. Three composite samples were made by thoroughly grinding weight ratios of 25% graphene with 75% ferrites. It is worth mentioning that these weight percentages (ferrite and graphene weight percentage) were chosen for preparing our composites according to different studies, which found that 25% of graphene and not less than 70% of ferrite can be an optimal percentage; this provided promising results for enhancing the electrochemical performance for different applications like supercapacitors, electromagnetic shielding, etc., [25,29,30,31]. Then, the composites were investigated by the EDX and SEM.

### 2.4. Magnetic and Dielectric Properties

The vibrating sample magnetometer (VSM) explored the ferrite samples’ magnetic properties and the composite samples. Frequency and temperature dependence of σ_AC_ and ε‘ were investigated for both ferrite and composite samples by using a lock-in amplifier, within a technique known as “complex impedance”, where the frequency ranged from 10^2^ to10^5^ Hz at different temperatures. In the setup of that technique, a small resistance R is connected in the series with the electrodes holding the sample. The current passing in the sample I can be calculated by dividing V_R_/R, where V_R_ is the voltage monitored across R using the lock-in amplifier. The applied frequency f of the used AC voltage V and the phase difference φ between the applied V and V_R_ are recorded as well. The AC conductivity σ_AC_ and the dielectric constant ε‘ can be calculated using well-known and published equations [32].

## 3. Results and Discussion

### 3.1. Characterization

**Figure 1:**
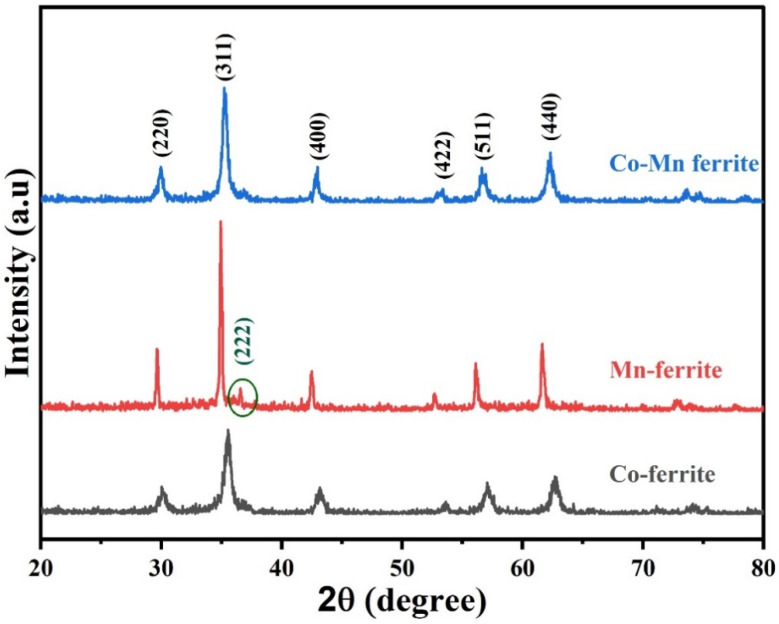
XRD patterns of the prepared ferrite samples.

The obtained typical patterns, well known for ferrites, ensure the formation of a single phase of the desired ferrites without any unwanted phases or residuals of constituent oxides or chlorides. The shown diffraction peaks presented in Figure 1 are attributed to (220), (311), (400), (422), (511), and (440) main planes known for the cubic spinel structure of ferrites [33,34]. In addition, the plane (222) is observed in the Mn-ferrite in agreement with the literature [35].

The crystallite sizes calculated from the XRD patterns (by the program associated with the equipment) are displayed in Table 1. In all samples, they were found within the nano range (10–70 nm). The Mn-ferrite sample shows the largest average crystallite size. We suggest that it may be due to a high-temperature exothermic reaction during the formation of the ferrite since it is well known that rising temperature during or after preparation leads to the enhancement of growth of the crystallites [36].

**Figure 2:**
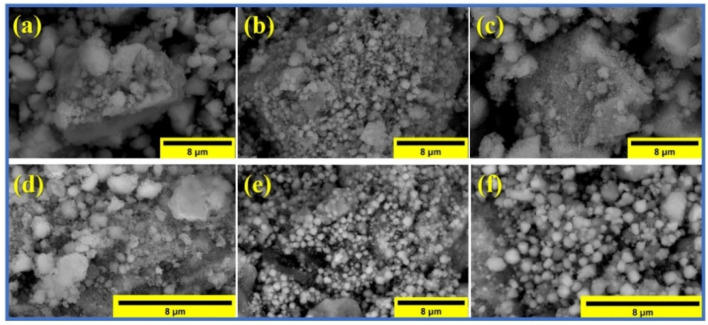
SEM images: Co-ferrite (**a**), Mn-ferrite (**b**), Co-Mn ferrite (**c**), Co-ferrite/graphene composite (**d**), Mn-ferrite/graphene composite (**e**), and Co-Mn ferrite/graphene composite (**f**).

The SEM images of the samples are shown in Figure 2a–f, and the average particle sizes of the ferrite samples calculated from the SEM images are presented in Table 1. It can be seen that they are in fair agreement with the average crystallite sizes calculated from the XRD data.

The graphene in the composite samples appeared in the images (d), (e), and (f).

**Figure 3:**
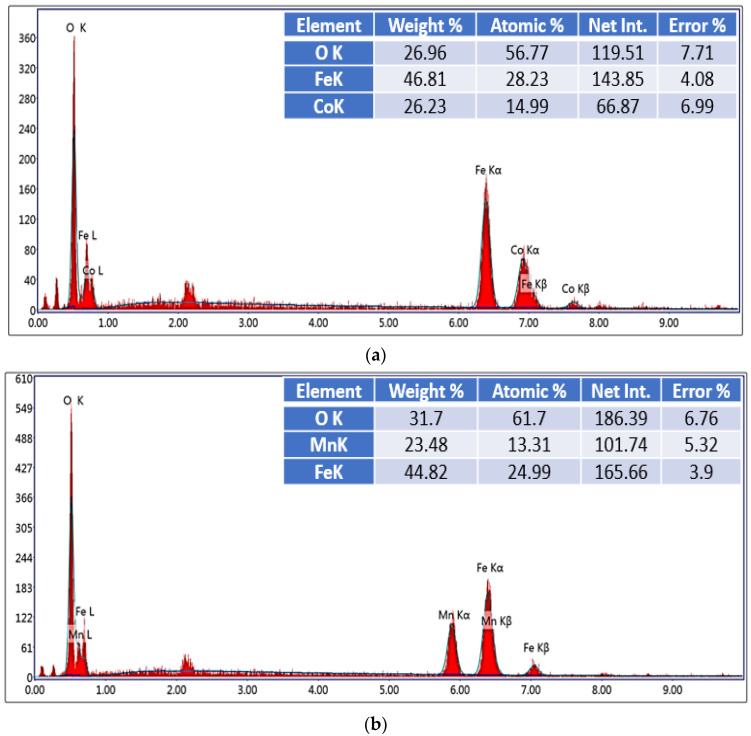

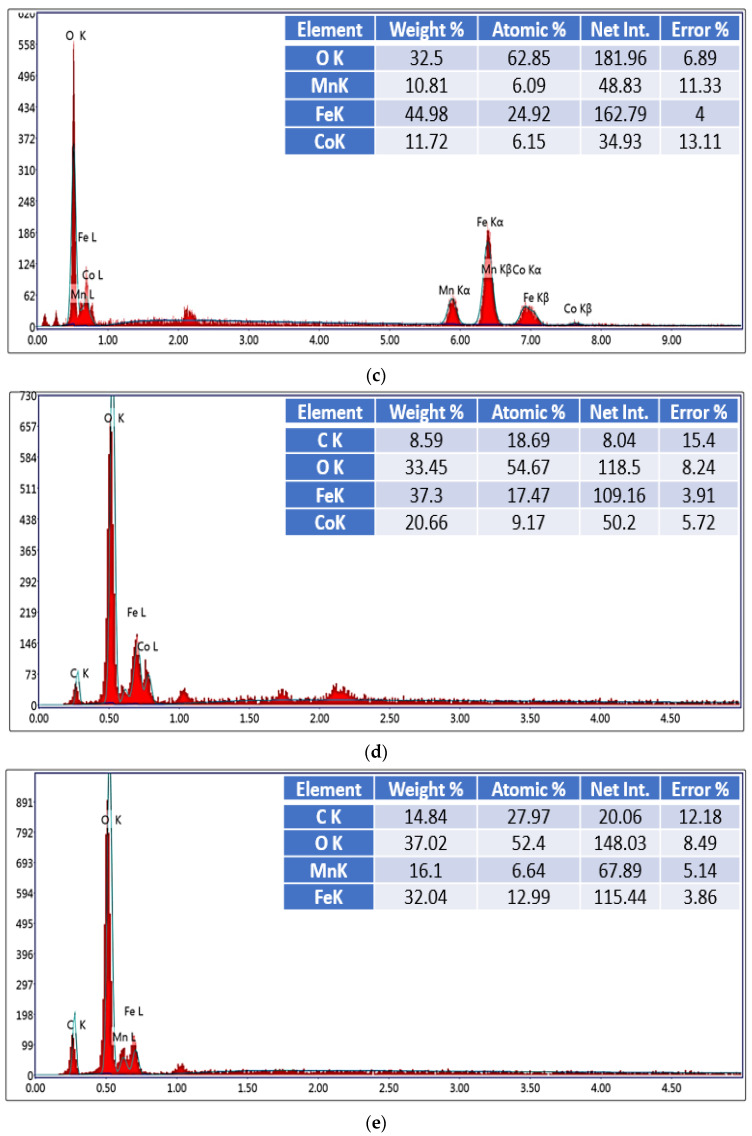

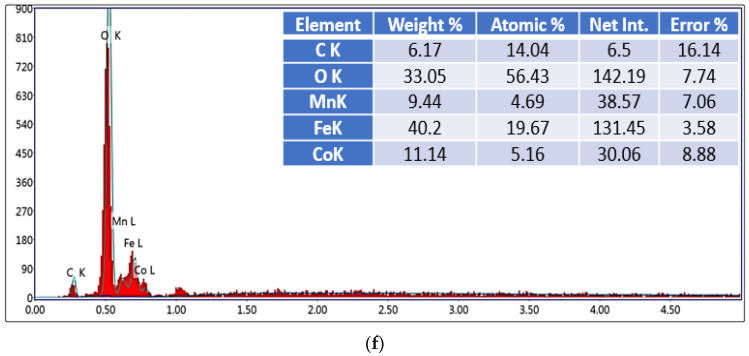
(**a**–**f**). EDX pattern of the Co-Mn ferrite/graphene composites.

For more composition confirmation, the EDX was used. The EDX patterns and tables are shown in Figure 3a–f, confirming the proper chemical compositions of the three prepared ferrite samples and the three composite samples with their intended elemental ratios.

### 3.2. VSM Measurements

**Figure 4:**
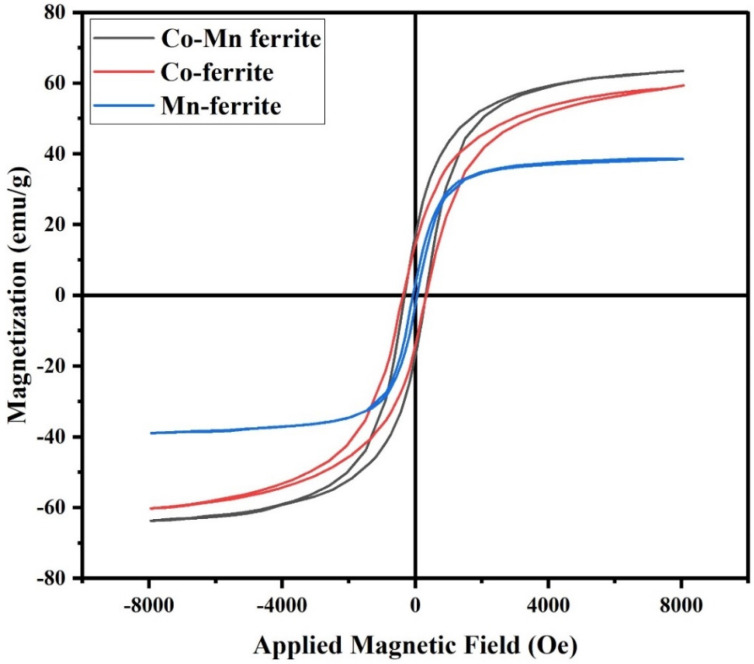
Magnetic hysteresis loops of the prepared ferrite samples.

**Figure 5:**
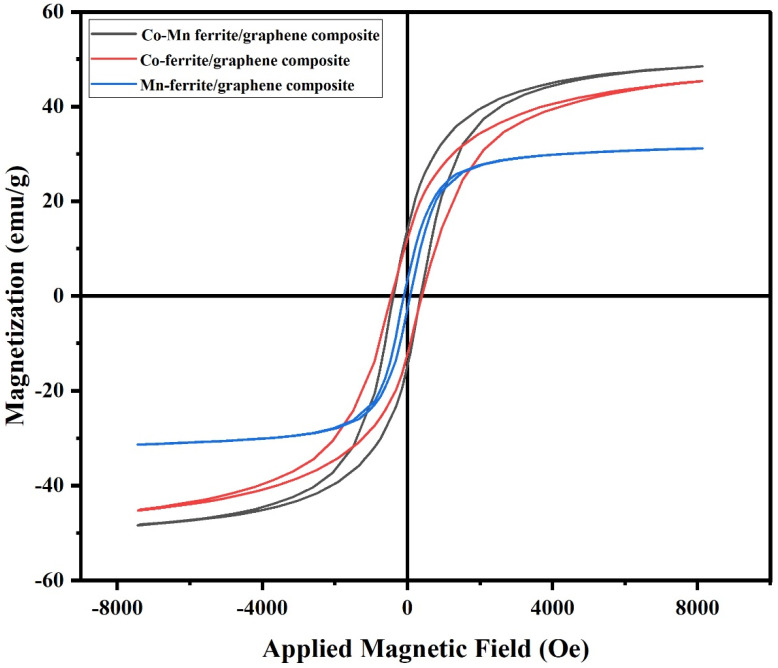
Magnetic hysteresis loops of the prepared ferrite/graphene composite.

The magnetic properties of the prepared nanoferrites and ferrite/graphene composites were investigated using a lab-built VSM [37]. The hysteresis loops plotted at room temperature are shown in Figure 4 and Figure 5.

The values of remnant magnetization M_r_, coercivity H_c_, and saturation magnetization M_s_ are recorded in Table 2.

It is observed that the saturation magnetization has the highest value for the Co-Mn ferrite sample whereas the lowest value is recorded for the MnFe_2_O_4_/graphene. Although the saturation magnetization M_s_ is actually due to the combination of many factors (extrinsic and intrinsic), such as chemical composition, grain size, and A-site and B-site ion exchange interactions [38], the main influencing factor is the cation distribution among the two sublattice sites A and B.

The way the vectors representing the spin magnetic moments are aligned, whether parallel, antiparallel, or canted, making angles with respect to each other, depends on the distribution of cations over these sublattices. M_s_ as a vector quantity is given by the sum of the magnetization vectors at the two sublattices, i.e., M_s_ = M_B_–M_A_.

According to the literature, Fe^3+^ ions have a strong preference to occupy octahedral B-sites. On the other hand, Co^2+^ ions may enter both A-sites and B-sites but with higher ratios in B-sites. Sanchez-Marcos et al. [39] and E. El-Ghazzawy [40] had suggested cation distributions based on the site preference of the ions, along with calculations of both experimental and theoretical magnetic moments, and they had reported values of M_s_ fairly close to ours.

It is noticed that the Mn-ferrite has a lower saturation magnetization than the cobalt ferrite and the mixed Co-Mn ferrite although the magnetic moments per ion for Co^2+^, Mn^2+^, and Fe^3+^ are 3 μ_B_, 5 μ_B_, and 5 μ_B_ respectively. This is because Mn is well known from the literature to be distributed almost evenly on the two sites, and the magnetic moments may cancel each other to a great extent. For example, D. Makovec et al. [41] prepared the structure of Mn_0.5_Zn_0.5_Fe_2_O_4_ ferrite in the form of nanoparticles and reported “the distribution of the constituting cations over the two sublattices”. Many other researchers have reported the dual preference of Mn for the two sites.

The second factor that may affect the value of M_s_ is the particle size, where it is known that when the particle sizes for the same material become smaller, M_s_ values decrease due to increase of the surface to grain ratio; with nanoparticles, the relatively large surface contains canted or disordered spin moment vectors and prevents the grain spin moments from easy alignment along the field direction and, consequently, results in an observed decrease of the saturation magnetization [42]. Hence, this may be the reason behind the smaller values of M_s_ than the same composition of ferrites with bulk particle sizes found in the literature a decade earlier.

The figures show that the hysteresis curves are not wide, with a relatively small coercive magnetic field classifying the studied samples as soft magnetic materials. The small coercive force values permit easy magnetization and demagnetization with limited losses. This observation suggests that all samples may be suitable for magnetic applications conditioned by low energy losses [43].

Regarding H_c_ values, it is found that they range from 70−450 Oe. “H_c_ value lying in the range of few hundred Oersteds is a necessary condition for electromagnetic shielding materials”, F. Aen et al., previously said [44]. Therefore, our H_c_ values may indicate another promising application of the samples.

Finally, for the composites, the results of the VSM show that when the graphene was introduced, an expected decrease in the M_s_, H_c_, and M_r_ occurred but without complete elimination of the magnetic properties.

### 3.3. AC Measurements

**Figure 6:**
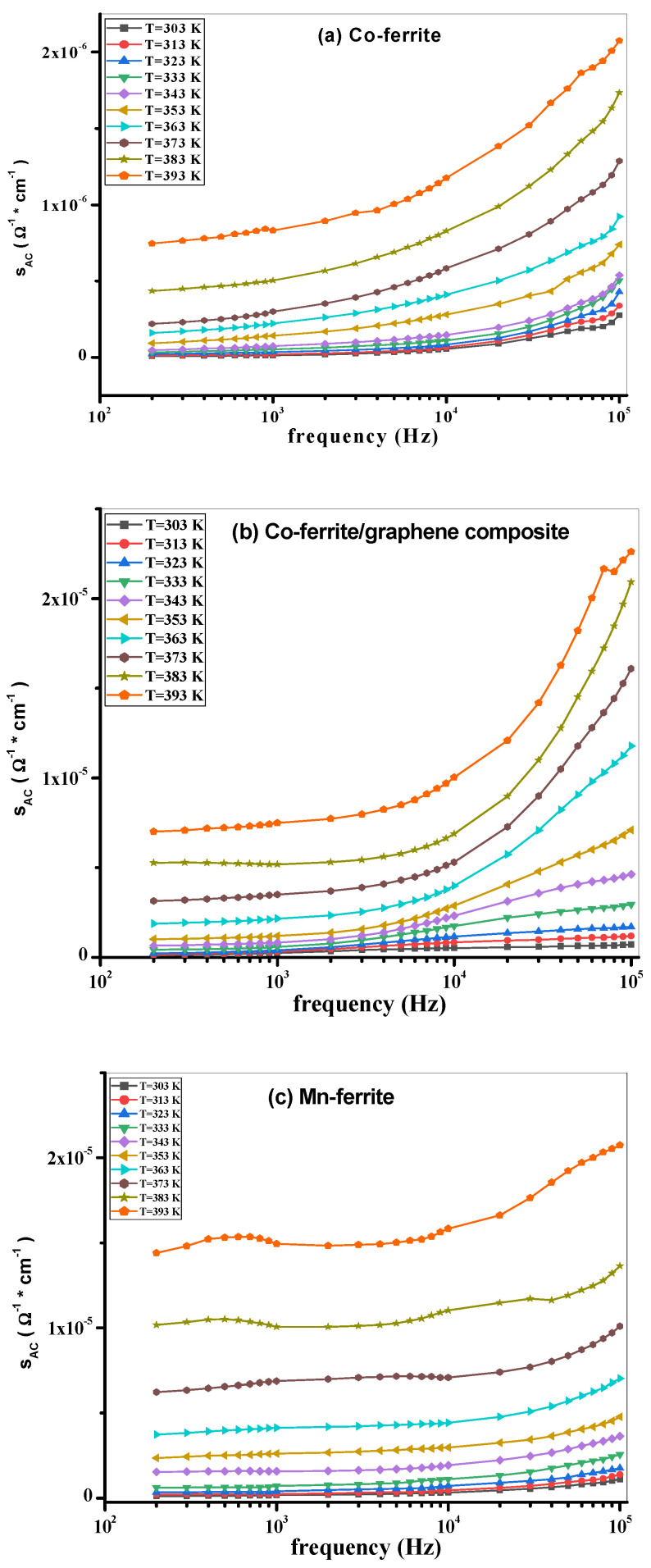

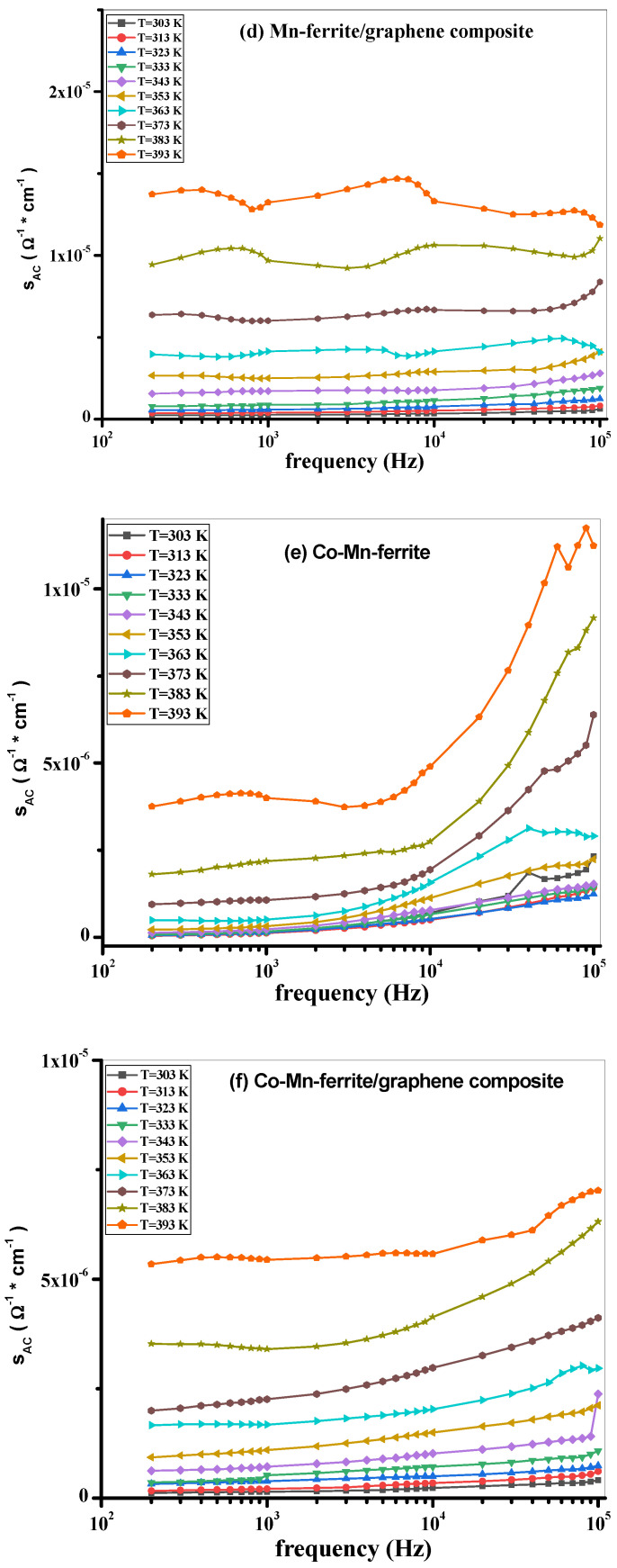
(**a**) σ_AC_ vs. frequency at different temperatures of Co-ferrite, (**b**) Co-ferrite/graphene, (**c**) Mn-ferrite, (**d**) Mn-ferrite/graphene, (**e**) Co-Mn ferrite, and (**f**) Co-Mn ferrite/graphene samples.

**Figure 7:**
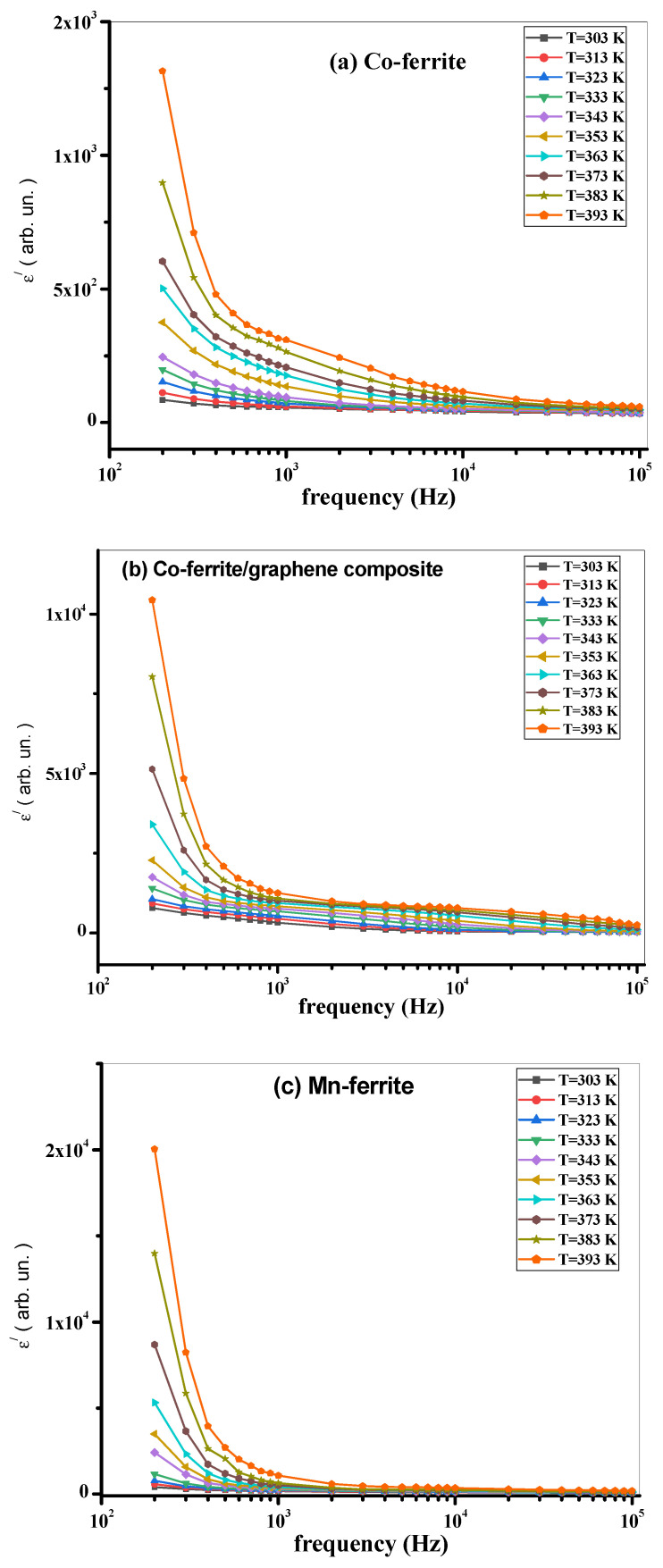

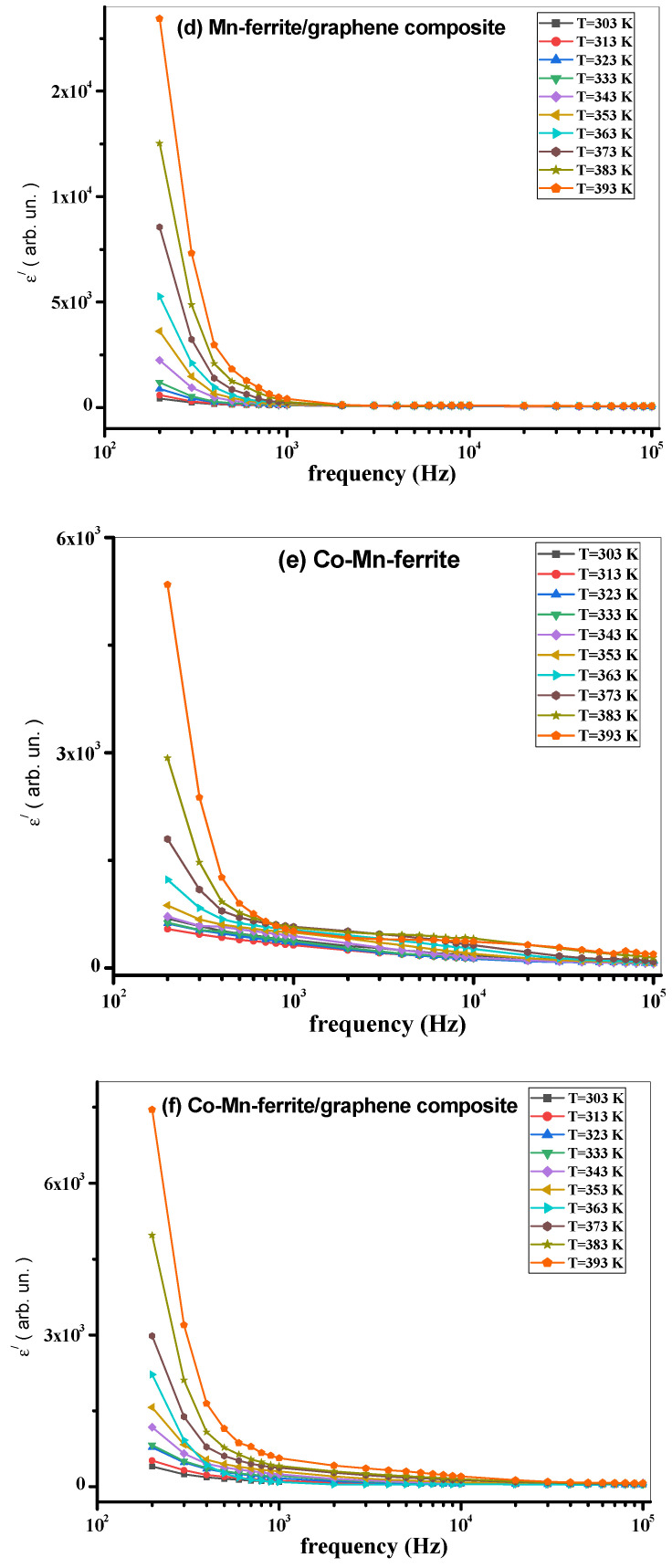
(**a**) ε‘ vs. frequency at different temperatures of Co-ferrite, (**b**) Co-ferrite/graphene, (**c**) Mn-ferrite, (**d**) Mn-ferrite/graphene, (**e**) Co-Mn ferrite, and (**f**) Co-Mn ferrite/graphene samples.

**Figure 8:**
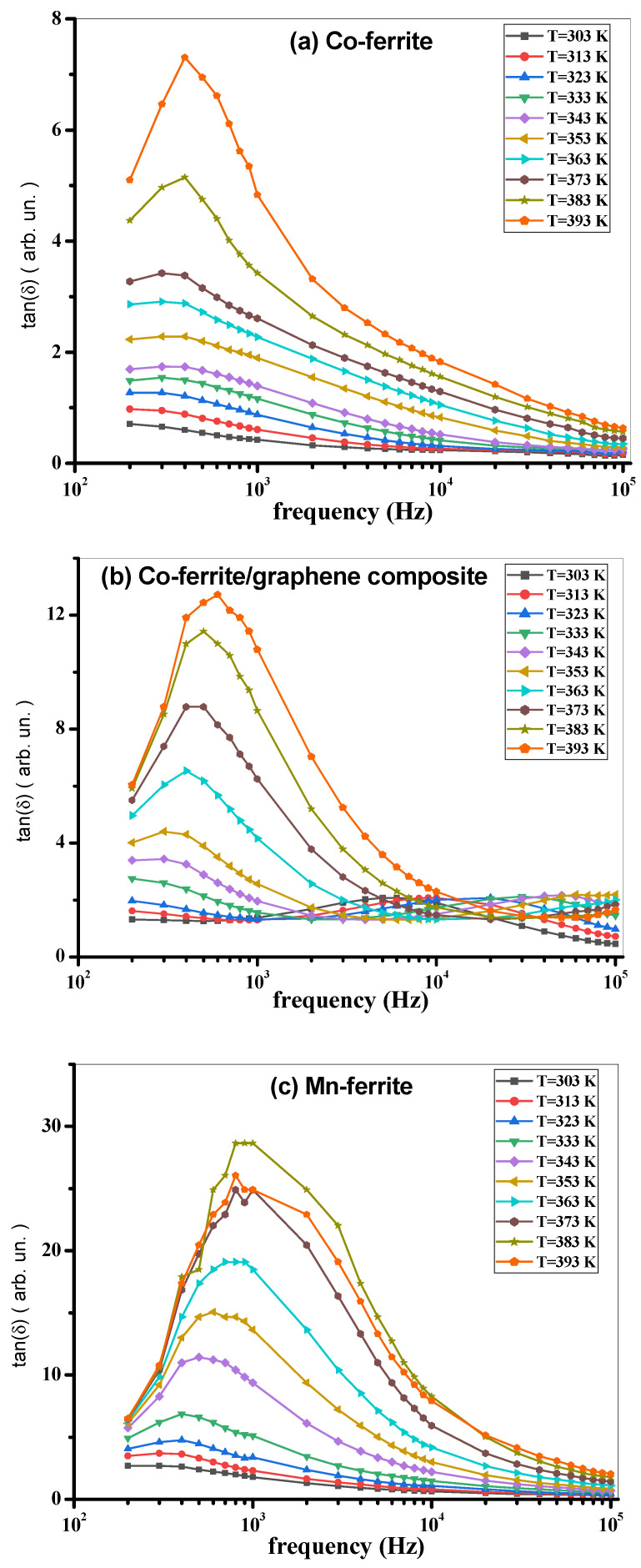

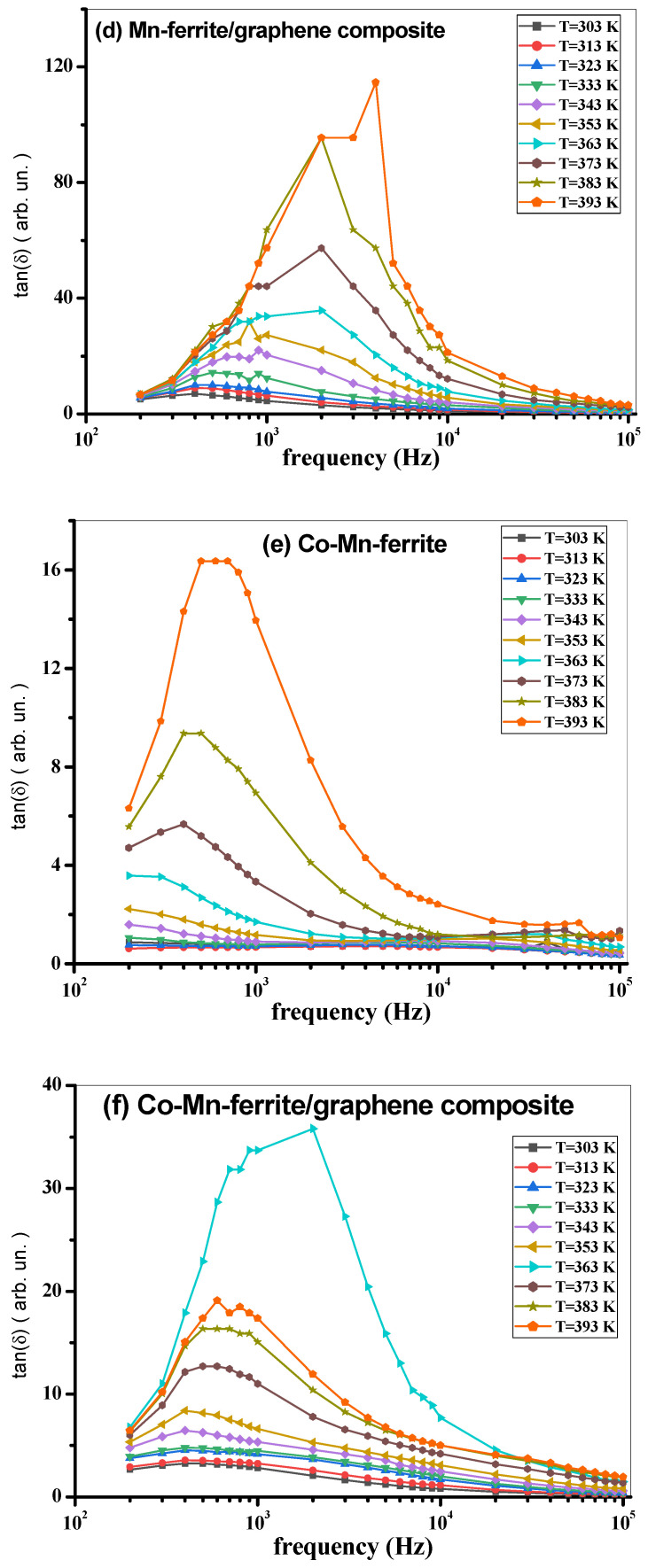
(**a**) tan(δ) vs. frequency at different temperatures of Co-ferrite, (**b**) Co-ferrite/graphene, (**c**) Mn-ferrite, (**d**) Mn-ferrite/graphene, (**e**) Co-Mn ferrite, and (**f**) Co-Mn ferrite/graphene samples.

To gain a larger insight of the properties of the present investigated samples, the AC conductivity σ_AC_, the dielectric constant ε‘, and the loss tangent tan(δ) were measured as functions of frequency and temperature, in the ranges of 200 Hz–100 kHz and 300 K–400 K, respectively. The σ_AC_, ε‘, and tan(δ) measurements are shown in Figure 6a–f, Figure 7a–f and Figure 8a–f, respectively.

The variation of σ_AC_ with temperature for all samples is similar and typically exhibits semiconducting behavior well known for ferrites [45], but regarding the values and order of magnitudes, it is obviously observed that introducing graphene enhanced the σ_AC_ conductivity values in the composite samples more than in the pure ferrite samples in agreement with the literature [14].

Furthermore, the observed frequency dependence of σ_AC_ is explained by a theoretical model proposed by Koops [46], who had described the heterogeneous structure of ferrites as consisting of fairly conducting grain regions separated by poorly conducting grain boundary regions. The AC conduction is accomplished by a hopping mechanism [47] of charge carriers between cations existing with different valences at the same site. Due to the difference in conductivity in the two regions, charge carriers reaching the grain boundary will pile up there, producing interfacial polarization. Moreover, the resistive grain boundaries are considered to be the dominant functional parts in conduction at lower frequencies, while the more conductive grains are the main contributor to conduction at higher frequencies. Moreover, electron hops can occur at lower temperatures while polaron hops can occur when the temperature rises. Therefore, in raising the temperature, the electron will move by the thermal activation, which can cause lattice distortion, resulting in a nonmonotonic behavior of AC conductivity and the other electrical characteristics [45].

Returning to the dielectric constant ε‘, it is well known that the dielectric properties of a material can arise from all or only some polarization mechanisms (space charge, orientational, ionic, and electronic). In the present work, through the measured frequency range of the applied field (200 Hz up to 100kHz), the dielectric relaxation that appears in Figure 7a–f arises from the ceasing of space charge polarization as the frequency is raised because the above-mentioned accumulated charge carriers at the boundaries cannot follow the variation of the field anymore. Then, ionic polarization ceases afterward. As shown in Figure 7a–f, ε‘ of the composite samples has higher values than those of the pure ferrites. This is easily explained based on a known assumption frequently mentioned in the literature [38] that the mechanism of polarization in ferrites is known to be the same as that of the conduction process, i.e., by hopping of charge carriers between multivalence ions of the same element. According to this argument, since the conductivity was enhanced by introducing the graphene, the dielectric constant is, therefore, expected to be increased, too.

On the other hand, the dielectric constant drops as the temperature lowers and rises as temperature increases. This behavior is due to the increase in thermal energy, which increases the charge carrier’s mobility [48]. Overall, graphene improves the dielectric constant values at low frequencies and high temperatures, and that is one of the main factors for the materials that can be used in manufacturing high-temperature ceramic supercapacitors [49].

Figure 8a–f shows the loss tangent for all the prepared samples. The term “dielectric loss tangent” refers to the amount of electrical energy dissipated in a material as a result of several physical processes, including domain wall dielectric resonance, electrical conduction, and dielectric relaxation [50,51]. Following Maxwell–Wagner interfacial polarization, all samples display the same loss tangent behavior. Low frequencies up to 10 kHz show an increase in the loss tangent, but as the frequency increases, the tangent steepens dramatically. Defects in the crystal lattice and dipoles cause this low-frequency response and significant interfacial polarization. Depending upon the prepared metal ferrite and the exact metal ferrite composition with graphene (composite samples), the loss tangent for all the prepared composites increases by adding the used graphene percentage due to the high electrical conductivity of graphene [30]. Sintering temperature and structural homogeneity, together with iron ion concentration, all have an effect on the tangent loss values [51]. It is worth mentioning that the hopping of electrons between Fe^2+^ and Fe^3+^ is regarded as the conduction mechanism in ferrites. As a result, the highest peak (observed peak) of the loss tangent may be observed when the hopping frequency is roughly equal to the frequency of the external applied electric field [52,53].

## 4. Conclusions

CoFe_2_O_4_, MnFe_2_O_4_, and Co_0.5_Mn_0.5_Fe_2_O_4_ nanosized particles were successfully synthesized by the coprecipitation method, and their composites with graphene were prepared with mixing weight ratios of 75% ferrite to 25% graphene. XRD and EDX analyses confirmed the formation of the desired ferrites with the intended composition ratios. The average particle sizes of ferrite nanoparticles calculated from the XRD are in strong agreement with those calculated from the SEM images. Magnetic properties were explored using the VSM for all ferrite and composite samples. The saturation magnetization values of ferrite nanoparticle samples are in agreement with the literature. On the other hand, the M_s_ values were lowered as expected for the composites but without eliminating the magnetic properties completely. The small coercive force values, which permit easy magnetization and demagnetization with little magnetic losses, suggest that all samples may be suitable for low magnetic losses applications. Moreover, according to the literature, the range of H_c_ values may be an indication of another promising application of the samples as electromagnetic shielding materials, which may stimulate future investigations for such application.

AC electrical properties were investigated for all the samples, such as the ferrites and ferrite/graphene composites, revealing their semiconducting nature as a function of temperature and the enhancement of AC conductivity and dielectric performance in the composite samples by adding the graphene, which may also be a good indication for using these composites in manufacturing supercapacitors.

## Figures and Tables

**Table 1:** Average particle size calculated from the XRD patterns and SEM images.

Composition	Calculated from All Peaks, (nm)	Calculated from the Maximum Peak, (nm)	Calculated from SEM Images, (nm)
CoFe_2_O_4_	20	13	19.2
CoMnFe_2_O_4_	25	16	18.8
MnFe_2_O_4_	75	64	55.5

**Table 2:** Remnant magnetization M_r_, coercivity H_c_, and saturation magnetization M_s_ of the investigated samples.

Composition	M_r_ (emu/g)	H_c_ (Oe)	M_s_ (emu/g)
CoMnFe_2_O_4_	17.0	325	63
CoFe_2_O_4_	14.5	350	60
MnFe_2_O_4_	3.5	70	39
CoMnFe_2_O_4/_graphene	14.0	390	48
CoFe_2_O_4_/graphene	12.5	450	45
MnFe_2_O_4_/graphene	3.5	100	31

## Data Availability

Data could be shared upon reasonable request.
